# Zinc Protects against Swine Barn Dust-Induced Cilia Slowing

**DOI:** 10.3390/biom14070843

**Published:** 2024-07-12

**Authors:** Christopher D. Bauer, Deanna D. Mosley, Derrick R. Samuelson, Jill A. Poole, Deandra R. Smith, Daren L. Knoell, Todd A. Wyatt

**Affiliations:** 1 Department of Internal Medicine, Division of Pulmonary, Critical Care & Sleep Medicine, University of Nebraska Medical Center, 985910 Nebraska Medical Center, Omaha, NE 68198, USA; christopher.bauer@unmc.edu (C.D.B.); deanna.mosley@unmc.edu (D.D.M.); derrick.samuelson@unmc.edu (D.R.S.); 2 Department of Internal Medicine, Division of Allergy & Immunology, University of Nebraska Medical Center, Omaha, NE 68198, USA; japoole@unmc.edu; 3 Department of Pharmacy Practice and Science, College of Pharmacy, University of Nebraska Medical Center, Omaha, NE 68198, USA; deandra.smith@unmc.edu (D.R.S.); daren.knoell@unmc.edu (D.L.K.); 4 Department of Environmental, Agricultural and Occupational Health, College of Public Health, University of Nebraska Medical Center, Omaha, NE 68198, USA; 5 Department of Veterans Affairs, Nebraska-Western Iowa Health Care System, Omaha, NE 68105, USA

**Keywords:** organic dust, cilia, zinc, lung

## Abstract

Agricultural workers exposed to organic dust from swine concentrated animal feeding operations (CAFOs) have increased chances of contracting chronic lung disease. Mucociliary clearance represents a first line of defense against inhaled dusts, but organic dust extracts (ODEs) from swine barns cause cilia slowing, leading to decreased bacterial clearance and increased lung inflammation. Because nutritional zinc deficiency is associated with chronic lung disease, we examined the role of zinc supplementation in ODE-mediated cilia slowing. Ciliated mouse tracheal epithelial cells were pretreated with 0–10 µg/mL ZinPro^TM^ for 1 h, followed by treatment with 5% ODE for 24 h. Cilia beat frequency (CBF) and protein kinase C epsilon (PKCε) activity were assayed. ODE treatment resulted in cilia slowing after 24 h, which was reversed with 0.5 and 1.0 µg/mL ZinPro pre-treatment. No zinc protection was observed at 50 ng/mL, and ciliated cells detached at high concentrations (100 µg/mL). ZinPro alone produced no changes in the baseline CBF and showed no toxicity to the cells at concentrations of up to 10 µg/mL. Pre-treatment with ZinPro inhibited ODE-stimulated PKCε activation in a dose-dependent manner. Based on ZinPro’s superior cell permeability compared to zinc salts, it may be therapeutically more effective at reversing ODE-mediated cilia slowing through a PKCε pathway. These data demonstrate that zinc supplementation may support the mucociliary transport apparatus in the protection of CAFO workers against dust-mediated chronic lung disease.

## 1. Introduction

Meat production in the United States involves large-scale concentrated animal feeding operations (CAFOs), requiring workers to be exposed to potentially hazardous air quality environments [1,2]. Each year, approximately 16,000 deaths are attributed to food production in the United States, with 80% being from animal production and producing livestock feed [3]. Workers in CAFOs are exposed to many possible respiratory components, such as ammonia, numerous gasses, including carbon dioxide and hydrogen sulfide, and organic dusts [1]. These organic dusts contain a variety of substances such as feed particles, fecal matter, animal dander, and fungal and bacterial components [4]. Livestock production is associated with an enhanced risk of respiratory symptoms. These symptoms range from rhinitis to chronic inflammatory lung disease [5]. Workers in CAFOs that are exposed to organic dusts have a higher incidence of lung disorders such as asthma, chronic bronchitis, and chronic obstructive pulmonary disease (COPD) [6,7]. Unfortunately, personal protective equipment (PPE) and/or respirators are not well utilized by CAFO workers due to many factors, such as overheating and comfort [8].

Exhalation and coughing are important to lung mechanical protection, but the first line of innate defense against inhaled dusts is mucociliary clearance [9]. We showed in an in vivo mouse model that the repetitive nasal delivery of organic dust extracts (ODEs) from swine barns results in a significant lung inflammatory pathology consisting of mononuclear infiltrates that aggregate in a peribronchiolar manner [10] in response to a sequential activation of the protein kinase C (PKC) pathway [11]. We have also shown that ODEs from swine barns cause cilia slowing [12] and that the slowing of cilia occurs through the activation of protein kinase C epsilon (PKCε) [13].

Zinc deficiency has been implicated in numerous lung diseases [14], representing a relatively common occurrence due to nutritionally inadequate diets [15]. We previously demonstrated that many agricultural workers who reported increased respiratory symptoms were also zinc-deficient [16]. Although zinc deficiency can be attributed to diet, inhibitors of zinc absorption also play a role. Phytate, which can be found in common foods such as cereal, rice, and corn, has a negative effect on zinc absorption [17]. To improve zinc absorption, the commercially available agent ZinPro^TM^ was developed, and it represents an uptake-enhanced, organic conjugate of zinc that is fed to livestock to assist in numerous functions, including immunity. Because nutrition impacts the exposome and zinc deficiency is associated with chronic lung disease, we hypothesized here that zinc supplementation could prevent ODE-mediated cilia slowing. To investigate this hypothesis, we investigated the role of ZinPro in the treatment of ODE-induced cilia slowing and the mechanistic regulation of ODE-induced epithelial cell PKCε. This study demonstrated that ZinPro treatment reversed ODE-induced cilia slowing by PKCε activity, suggesting that zinc supplementation may lower the risk of developing organic dust-mediated inflammatory lung disease.

## 2. Materials and Methods

### 2.1. Cell Culture

Both human and mouse lung epithelial cells were used. BEAS-2Bs are a non-tumorigenic human bronchial epithelial cell line and were obtained from the American Type Culture Collection (ATCC, Manassas, VA, USA). BEAS-2B cultures were grown at 37 °C with 5% CO_2_ in M-199 media. Cells were passaged by adding Gibco TrypLE Express (Life Technologies Corp., Grand Island, NY, USA) for 5–10 min and TrypLE Express inactivated with phosphate-buffered saline (PBS; pH 7.4) containing 10% fetal bovine serum (FBS) (R&D Systems, Pittsburgh, PA, USA). Collected cells were centrifuged for 10 min at 193× *g* (Beckman Coulter, Brea, CA, USA), and an additional wash was performed with PBS + fetal bovine serum (FBS). Cells were counted, plated, and allowed to proliferate to 80–90% confluence for subsequent assays.

Wild-type C57BL/6 mice (Jackson Laboratory, Bar Harbor, ME, USA) were euthanized, and their tracheas were removed and cut longitudinally to expose the inner lumen. The tracheas were incubated overnight at 4 °C in 1.5 mg/mL Pronase digestion buffer (Sigma Aldrich, St. Louis, MO, USA). Digestion was halted with 10% FBS, the tracheas were removed, and digestion buffer media were centrifuged at 193× g for 3 min to collect the cells. Mouse tracheal epithelial cells (MTECs) were resuspended in basic media (Gibco-Thermo Fisher, Waltham, MA, USA) with 10% FBS and placed in 60 mm tissue culture dishes. After the dishes were incubated for 3–4 h at 37 °C, the media were removed and centrifuged at 193× g for 3 min. MTECs were resuspended in an air–liquid interface (ALI) Cell Nutrient Mix consisting of MTEC basic media with growth supplements (Gibco-Thermo Fisher), counted, and then seeded onto cell culture inserts with 1 × 10^5^ cells per insert and returned to the incubator. Once the cells reached confluency, the media were removed, and the cells were exposed to air until cilia formed (approximately 14 d). For some assays, mouse ciliated tracheal rings [18] or detergent-extracted ciliary axonemes lacking a cell membrane were prepared as previously described [19].

### 2.2. Organic Dust Extract (ODE) Preparation

Aqueous organic dust extract (ODE) prepared from swine confinement feeding facilities was the exposure agent used in these experiments. Briefly, settled surface dust (1 g) was incubated in sterile Hank’s Balanced Salt Solution (10 mL; Mediatech, Manassas, VA, USA) for 1 h and centrifuged twice for 30 min at 2850× *g*, with the final supernatant filter-sterilized (0.22 μm) to remove microorganisms and coarse particles. Stock ODE (100%) was batch-prepared and stored at −20 °C; aliquots were diluted for each experiment to a final concentration (vol/vol) of 5% in sterile phosphate-buffered saline (PBS, pH 7.4; diluent) for in vitro exposures and 12.5% for in vivo studies. A semiquantitative analysis by means of inductively coupled plasma mass spectrometry revealed the presence of the metals B, Mg, Ti, Mn, Fe, Co, Ni, Cu, Rb, Mo, and Zn (in 100% dust concentrate: B, 1380 ng/mL; Mg, 144,600 ng/mL; Ti, 1166 ng/mL; Mn, 275.5 ng/mL; Fe, 4226 ng/mL; Co, 59.7 ng/mL; Ni, 371 ng/mL; Cu, 3295.5 ng/mL; Rb, 1076.5 ng/mL; Mo, 132 ng/mL; Zn, 8797.5 ng/mL) [20]. Endotoxin concentrations from the prepared ODE were determined using limulus amebocyte lysate assay (Lonza, Walkersville, MD, USA). Endotoxin levels averaged 5.232–10.464 mg (~40–200 EU) for 100% ODE. Mass spectrometry studies of ODE performed previously revealed significant amounts of muramic acid (a peptidoglycan marker) and 3-hydroxy fatty acids (a endotoxin marker), but not ergosterol (a fungi marker), as compared to house dust [21,22].

### 2.3. In Vivo Mouse Intranasal Inoculation and Infection

Mice were lightly anesthetized using isoflurane and received 50 µL of 12.5% ODE intranasally and daily (excluding weekends) for 13 days. Some mice were infected by oropharyngeal aspiration with 100 µL of 4 × 10^8^ colony-forming units (CFUs) per mouse of *Streptococcus pneumoniae* (D39 strain) following ODE treatments. The mice were then euthanized 48 h later to collect total lung CFUs. All animal experiments were evaluated and approved by the University of Nebraska Medical Center Institutional Animal Care and Use Committee (Protocol #22-070-11-FC).

### 2.4. Determination of Colony-Forming Units (CFUs)

Whole lungs were removed from the euthanized mice and placed in 800 µL of PBS. The lungs were then homogenized using a whole tissue homogenizer (Dremel 300, Mt. Prospect, IL, USA). Serial dilutions were performed on the lung homogenate and then plated onto blood agar plates (Remel, Lenexa, KS, USA). Plates were incubated overnight at 37 °C with 5% CO_2_. Plates were removed the following day, and bacterial colonies were counted to determine total lung CFUs.

### 2.5. Live Animal MicroCT

Experimental mice were evaluated for changes in lung density using live animal microCT scanning. Mice were rendered unconscious using an RAS4 anesthesia device (Perkin Elmer, Waltham, MA, USA) with the following settings: 1.5 induction chamber, 3.5 CT scanner, exhaust 1.5, and isoflurane set to level 3. Mice were exposed to vaporized isoflurane for a maximum of 3 min. Each mouse was transferred and positioned supine on the scanning bed of a Quantum GX-2 microCT scanner (Perkin Elmer, Waltham, MA, USA). A nose cone attachment was used to keep the animal unconscious, with continuous isoflurane exposure during scanning. Using the Quantum GX2’s respiratory gating function, a high-speed, 4 min lung gating scan was recorded for lung assessment. The microCT scanning parameters were as follows: 50 kV, 114 µA, and 36 FOV mm. A Cu 0.06 + Al 0.5 X-ray filter was used, and the total radiation dose incurred by each mouse was 338 mGy per scan. A baseline scan was performed on each mouse. Following the baseline scan, the mice received sterile PBS or 12.5% ODE daily. After 13 intranasal treatments, as described above, the mice were re-scanned to determine the damage caused by ODE. Once scanning was completed, 3D models of the lung scans were constructed using the open-source software 3D Slicer (version 4.11) and analyzed to quantify the extent of lung damage, comparing post-exposure to baseline determinations for each mouse.

### 2.6. Measurement of Tissue Zinc Content

Whole lungs from mice repetitively nasal-instilled with either sterile PBS or 12.5% ODE were collected and flash frozen until assayed. Lung samples were thawed and dried using a vacuum oven, then digested in 1 mL of Nitric Acid and Perchloric Acid (1:2) for 2 h at 80 °C. Zn measurements were analyzed using a PinAAcle 500 Atomic Absorption Spectrometer (Perkin Elmer). Results were standardized by total weight (g) assayed.

### 2.7. Cilia Beat Frequency Assay

A detailed characterization of the Sisson–Ammons Video Analysis (SAVA) system can be found in [23]. SAVA uses whole-field analysis to analyze cilia beat frequency (CBF) while eliminating operator bias due to the selection of areas containing aberrantly fast or slow-moving cilia. SAVA was used to detect regions of ciliated wild-type MTECs that were treated with or without 5% ODE and with or without 1–10 µg/mL ZinPro. ZinPro is a form of zinc that is conjugated to various amino acids for the purpose of enhancing its cellular uptake and bioavailability in vivo. The ZinPro Corporation (Minneapolis, MN, USA) kindly provided the reagent used in this study, which was in the conjugate form of glutamic acid and lysine (1:1). The changes in CBF and total number of motile points were measured at multiple time points over 24 h.

Some ciliated MTECs were fed basally with Chelex-treated zinc-free FBS containing media for 72 h. Although the media were zinc free, they did contain other essential cations (Ca++, Mg++, etc.). Our medium source was assayed through the Atomic Absorption Spectrum, and no contaminating zinc was discovered. After zinc chelation, MTECs were pretreated for 4 h with 10 µM N,N,N′,N′-Tetrakis-2-pyridylmethylethylenediamine (TPEN) (Sigma Aldrich) to chelate intracellular zinc. Following the 4 h preincubation, TPEN was removed from the media. The cells were then pretreated with ZinPro (1 µg/mL) for 1 h, followed by 5% ODE, and CBF was measured 24 h later.

### 2.8. Kinase Activity Assay

BEAS-2B cells at 80% confluency were pretreated for 1 h with or without ZinPro. Following pretreatment, 5% ODE was added to the cells and incubated at 37 °C at 5% CO_2_ for 1 h, 6 h, or 24 h. The cells were flash frozen with liquid N_2_ in 250 µL of a cell lysis buffer containing 35 mM 1M Tris-HCl (pH 7.4), 0.4 mM EGTA, 10 mM MgCl_2_, 10 mM phenylmethylsulfonyl fluoride, and a 1:100 dilution of Sigma Protease Inhibitor Cocktail (SPIC, Sigma-Aldrich, St. Louis, MO, USA). The cells were scraped, transferred into microfuge tubes, and dissociated by sonication for 5 sec each. Protein (PKCε) catalytic activity was determined using the method of Hannun et al. [24]. Equal volumes (20 µL) of radiolabeled ATP (10 µCi/mL [γ-^32^P]-ATP; Revvity Health Sciences, Waltham, MA, USA) and PKC reaction mix (containing 24 µg/mL phorbol 12-myristate 13-acetate, 30 mM dithiotreitol, 150 µM adenosine triphosphate, 45 mM magnesium acetate, and PKC epsilon-specific substrate peptide (Bachem, Torrance, CA, USA) mixed in a 50 mM Tris-HCl buffer) were dispensed into 12 × 75 mm glass tubes. A protein sample (20 µL) was added to each tube in a 30 °C water bath, and each tube was incubated for exactly 10 min. The reaction was halted by adding 50 µL from each tube to be spotted onto Phosphocellulose exchange papers (St. Vincent’s Institute of Medical Research, Fitzroy, Australia), and these were submerged in 85% phosphoric acid immediately.

For some experiments, MTECs were incubated for 1 h with or without the intracellular zinc-chelating agent Tris(2-pyridylmethyl)amine (TPA) (Sigma-Aldrich). Following the 1 h preincubation, TPA was removed from the media. The cells were then pretreated with ZinPro (1 µg/mL) for 1 h, followed by 5% ODE. After 24 h, 250 µL PBS was added to the ALI cell culture inserts, and the cells were harvested. The cells were then centrifuged at 200× *g* for 10 min, and the supernatant was discarded. The cells were resuspended in 250 µL of cell lysis buffer and flash frozen in liquid N_2_ to be assayed for PKC activity. For cell-free in vitro enzyme inhibition assays, ZinPro or amino acids (lysine/glutamine; 1:1) were added at various concentrations to purified PKCε, and a PKC activity assay was performed. Negative controls consisted of the absence of an enzyme or substrate.

### 2.9. Statistical Analysis

Data are presented as the mean ± standard deviation (SD) with scatter plots depicted for each data point. Student’s *t*-test was used to assess the statistical difference between two groups, and a one-way analysis of variance (ANOVA) was used to assess statistical differences among three or more experimental groups, with Tukey’s post hoc test for multiple comparisons between any two groups. Statistical significance was accepted at a *p* value < 0.05. The determination of data normality and statistical analyses were performed using GraphPad Prism 10 (San Diego, CA, USA) software.

## 3. Results

### 3.1. Repetitive Organic Dust Extract (ODE) Treatment Increases Lung Inflammation and Decreases Bacterial Clearance

**Figure 1:**
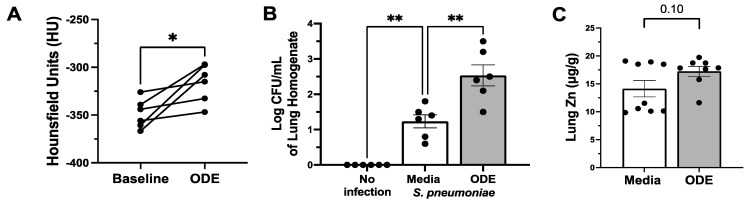
Repetitive nasal instillation of organic dust extract (ODE) increases lung inflammation and decreases bacterial clearance. (**A**) Live animal microCT of the lung showing density in Hounsfield units (HUs) in mice at baseline followed by 3 weeks of 12.5% ODE (* *p* < 0.02, paired *t*-test of baseline vs. ODE for each mouse, n = 6). (**B**) *S. pneumoniae* lung burden at 48 h post infection in 12.5% ODE-treated mice (** *p* < 0.001, n = 6/group). (**C**) Total lung zinc content in mice repetitively instilled with sterile PBS or 12.5% ODE (n = 10).

Previous studies have shown a distinct inflammatory histopathology of lymphoid aggregates in the lung of mice nasally instilled with 12.5% ODE daily over 3 weeks [25]. To determine lung density as a measure of inflammation, we performed live animal microCT scans on uninfected mice before and after 3-week repetitive ODE exposure. ODE treatment significantly (*p* < 0.02) increased lung density, as measured by Hounsfield units (a representation of lung inflammation) in the mice (Figure 1A). The mice repetitively exposed to ODE also demonstrated a significant increase in (*p* < 0.001) bacterial burden 48 h after infection with *S. pneumoniae* (Figure 1B). These effects were not due to an alteration in tissue zinc content in response to ODE, as the ODE-treated mice showed no differences in whole-lung zinc deficiency compared to the control mice nasally instilled with sterile PBS (Figure 1C).

### 3.2. ODE-Induced Slowing of Ciliary Beat Frequency (CBF) in Mouse Tracheal Epithelial Cells (MTECs) Is Prevented by Pretreatment with ZinPro

**Figure 2:**
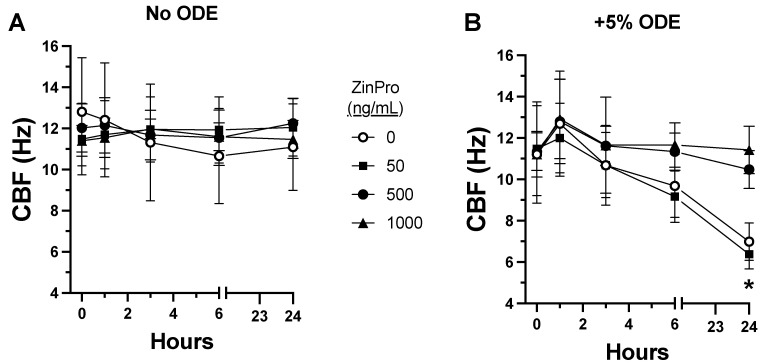
ODE-induced slowing of ciliary beat frequency (CBF) in mouse tracheal epithelial cells (MTECs) is prevented by pretreatment with ZinPro. (**A**) Time course (0–24 h) of MTECs treated with 0–1 µg/mL ZinPro shows no effect on baseline CBF in Hertz (Hz). (**B**) Time course in the presence of 5% ODE (* *p <* 0.01 0–50 ng/mL vs. 500 ng/mL^−1^ µg/mL ZinPro; one-way ANOVA, n = 20).

To further explore the cause of defective bacterial clearance in the ODE-treated mice, we measured ciliary beating in primary mouse tracheal epithelial cells (MTECs) cultured in a polarized state to express active motile cilia. MTECs were treated with increasing concentrations (50 ng/mL to 1 µg/mL) of ZinPro^®^, an enhanced amino acid and zinc conjugate, and CBF was assessed out to 24 h. There was no effect on the CBF between the baseline media control and ZinPro over time (Figure 2A). However, at very high concentrations of ZinPro (0.1–1 mg/mL), ciliated cell detachment and cytotoxicity were observed. As previously reported [12], 5% ODE significantly decreased (*p* < 0.01) the CBF through a 24 h treatment; however, ODE-induced cilia slowing was prevented when MTECs were pretreated for 1 h with ZinPro at concentrations of 0.5 and 1.0 µg/mL (Figure 2B), whereas ZinPro concentrations below 0.5 µg/mL did not reverse ODE-induced cilia slowing.

### 3.3. ODE-Induced Activation of Protein Kinase C Epsilon (PKCε) in Mouse Tracheal Epithelial Cells (MTECs) Is Prevented by Pretreatment with ZinPro

**Figure 3:**
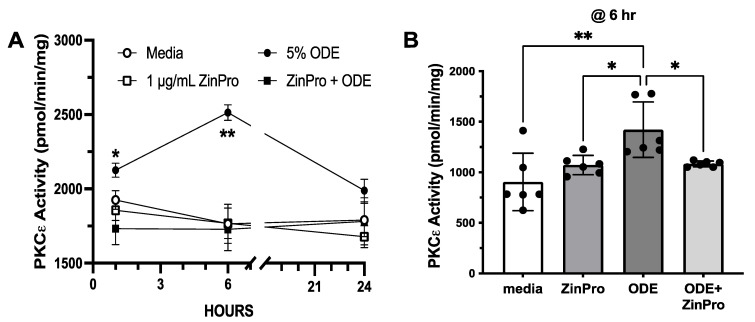
ODE-induced activation of protein kinase C epsilon (PKCε) in mouse tracheal epithelial cells (MTECs) is prevented by pretreatment with ZinPro. (**A**) Time course (0–24 h) of MTECs treated with 1 µg/mL ZinPro in the presence or absence of 5% ODE (* *p <* 0.02 at 1 h ODE vs. media control; ** *p <* 0.003 at 6 h ODE vs. media control). (**B**) PKCε activity at 6 h with ±5% ODE and ±500 ng/mL ZinPro (* *p <* 0.01; ** *p* < 0.001; one-way ANOVA, n = 9).

The activation of PKCε results in decreased ciliary beating [13,26]. Likewise, 5% ODE activates PKCε, beginning after 1 h, with the maximum kinase activation occurring within 6 h, followed by kinase autodownregulation at 24 h (Figure 3A). PKCε activation and subsequent autodownregulation are prevented when MTECs are pretreated for 1 h with 1 µg/mL Zinpro (Figure 3A). Moreover, 0.5 µg/mL ZinPro (the lowest concentration) was also capable of significantly (*p* < 0.01) blocking ODE-stimulated PKCε at 6 h or the peak activity time (Figure 3B).

### 3.4. Intracellular Zinc Chelation Inhibits ZinPro Reversal of ODE-Induced Cilia Slowing

**Figure 4:**
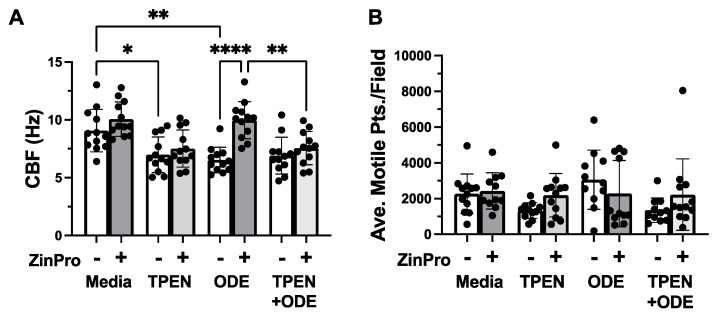
Intracellular chelation of zinc blocks ZinPro reversal of ODE-induced cilia slowing. (**A**) Ciliated MTECs were pretreated for 4 h with or without 5 µM TPEN (N,N,N′,N′-Tetrakis-2-pyridylmethylethylenediamine), followed by 24 h of ±1 µg/mL ZinPro and ±5% ODE (* *p <* 0.01, ** *p <* 0.001, **** *p <* 0.0001 one-way ANOVA, n = 20). (**B**) No significant differences in the total number of moving cilia were observed with Chelexed media or TPEN treatment at the time of recording (24 h).

In order to determine whether the restoration of the CBF was specifically due to zinc, we examined the impact of the intracellular chelator N,N,N′,N′-Tetrakis-2-pyridylmethylethylenediamine (TPEN). All media were initially depleted of zinc using Chelex. MTECs were pretreated with or without 5 µM TPEN for 4 h prior to exposure to 5% ODE in the presence or absence of 1 µg/mL ZinPro, and the CBF was assayed at 24 h. TPEN blocked the ability of ZinPro to rescue ODE-induced cilia slowing (Figure 4A). ZinPro alone did not change the baseline CBF media controls, but, as before, it prevented ODE-mediated cilia slowing. TPEN alone produced a less robust but significant (*p* < 0.01) decrease in the CBF compared to the media controls but showed no difference in the presence of ODE. No toxicity was observed with TPEN or Chelexed media and no significant differences in the total number of motile cilia were observed under any treatment conditions (Figure 4A).

### 3.5. Intracellular Chelation of Zinc Blocks ZinPro Reversal of ODE-Induced PKCε Activation

**Figure 5:**
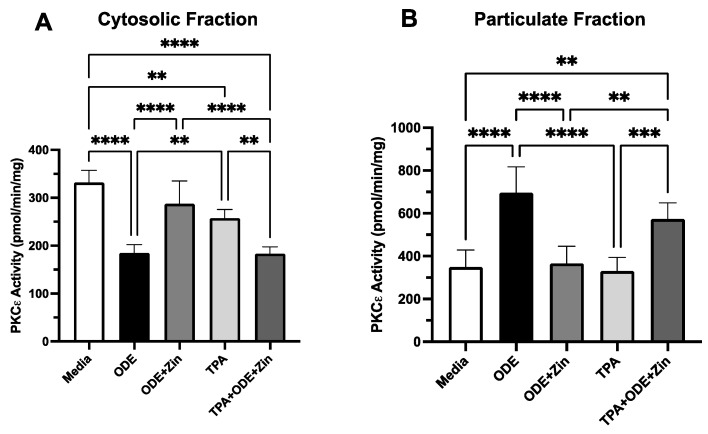
Intracellular chelation of zinc blocks ZinPro reversal of ODE-induced PKCε activation. Ciliated MTECs were pretreated for 1 h with or without 5 µM TPA (Tris(2-pyridylmethyl)amine) followed by 24 h of ±1 µg/mL ZinPro and ±5% ODE. (**A**) Cytosolic cell fraction measuring non-translocated PKC (** *p <* 0.001, **** *p <* 0.0001 one-way ANOVA). (**B**) Particulate cell fraction showing translocation-activated PKC (** *p <* 0.001, *** *p <* 0.005, **** *p <* 0.0001 one-way ANOVA, n = 9).

The impact of the intracellular sequestration of zinc was then examined with regard to the ZinPro’s effect on PKCε activation. MTECs were pretreated similarly to TPEN, as described above but using the intracellular zinc chelator Tris(2-pyridylmethyl)amine (TPA) for 1 h, and cells were then fractionated into a non-translocated (inactive) cytosolic fraction and translocated (active) particulate PKCε fractions. The peak kinase activity was measured at 6 h following cell treatment and exhibited a significant ODE-mediated decrease (indicating translocation) in cytosolic PKCε, which was prevented by 1 µg/mL ZinPro (Figure 5A). The beneficial effect of ZinPro was reversed by TPA, confirming the results seen for TPEN. This coincided with the reversal of the translocated enzyme particulate fraction (Figure 5B), showing mechanistic agreement between PKCε activity and cilia slowing.

### 3.6. Zinc Inhibits In Vitro Protein Kinase C Epsilon (PKCε) Activity

**Figure 6:**
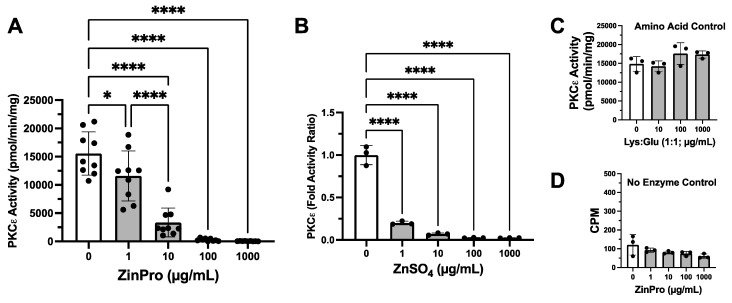
Zinc inhibits in vitro protein kinase C epsilon (PKCε) activity. (**A**) Purified PKCε treated with 0–1 mg/mL ZinPro in vitro (* *p <* 0.05; **** *p <* 0.0001, one-way ANOVA, n = 12). (**B**) Purified PKCε treated with 0–1 mg/mL ZnSO_4_ in vitro (**** *p <* 0.0001, one-way ANOVA, n = 9). (**C**) Purified PKCε treated with 0–1 mg/mL lysine and glutamic acid in vitro as a control. (**D**) Substrate-only negative control treated with 0–1 mg/mL ZinPro.

To determine if these observations are due to a direct effect of zinc on PKCε, in vitro enzyme activity assays were conducted using a cell-free purified enzyme and substrate. When 0.1 µg/µL purified PKCε was co-incubated with 0–1 mg/mL ZinPro in vitro, a significant (*p* < 0.05) concentration-dependent inhibition of phosphate transfer to substrate peptide was observed (Figure 6A). Similarly, purified PKCε treated with 0–1 mg/mL ZnSO_4_ in vitro also significantly (*p* < 0.0001) inhibited the ability to phosphorylate its substrate (Figure 6B). As a control to account for the amino acid conjugates attached to ZinPro, purified PKCε treated with the same amount (0–1 mg/mL) of lysine and glutamic acid in vitro did not inhibit kinase activity (Figure 6C). As anticipated, the substrate-only negative control showed no changes in non-specific phosphate incorporation by ZinPro (Figure 6D).

### 3.7. CBF Is Enhanced in Isolated Ciliary Axonemes but Not Intact Mouse Tracheal Cells or Tissue

**Figure 7:**
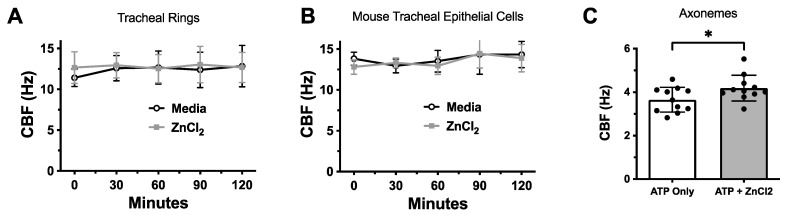
CBF is enhanced in isolated ciliary axonemes but not intact mouse tracheal cells or tissue. (**A**) Freshly excised mouse tracheal rings with beating cilia treated with ±10 µM ZnCl_2_. (**B**) Ciliated MTEC treated with ±10 µM ZnCl_2_. (**C**) De-membranated isolated ciliary axonemes activated with 1 mM ATP and treated with ±10 µM ZnCl_2_. (* *p <* 0.03 unpaired *t*-test, n = 12).

Knowing that ZinPro and zinc sulfate directly inhibit cell-free PKCε in vitro, we compared whether the ability of ZinPro to restore the CBF in situ was superior to another commonly used zinc salt form: zinc chloride. The treatment of intact ciliated mouse tracheal tissue rings (Figure 7A) or isolated ciliated polarized MTEC ALI cultures (Figure 7B) with ZnCl_2_ at doses of up to 10 µM had no impact on the CBF. However, isolated and de-membranated ciliary axonemes that were activated by 1 mM ATP to bend in culture exhibited a small but significant (*p* < 0.03) increase in motility (Figure 7C), further suggesting that the amino acid-coupled active transport of ZinPro leads to enhanced intracellular zinc uptake when compared to conventional zinc salt forms (ZnCl_2_ or ZnSO_4_).

## 4. Discussion

Our studies demonstrated that the uptake-enhanced, highly cell-permeable zinc delivery agent ZinPro sufficiently reversed ODE-induced cilia slowing through modulating PKCε activity in airway epithelial cells. The circulating zinc levels in healthy male adults are approximately 12 µmol/L [27]. Concentrations of ZinPro of 0.5–1.0 µg/mL, as utilized in our experiments, would equate to the supplementation of 10–15 µmol/L of ZinPro for translational application. As prior studies have demonstrated that repetitive ODE treatment induces lung inflammatory pathology consisting of lymphoid aggregates [25], this current work extends these observations by live-animal micro-CT imaging, with increased inflammation represented by increases in density measurements (Hounsfield units). The ZinPro’s effect on ciliary motility was ascribed to zinc’s properties (as opposed to its conjugates), as ZinPro’s positive effects were abrogated in the presence of several zinc chelators. Moreover, zinc directly affects the ciliary beat of axonemes alone. Thus, these data suggest that supplementation with zinc may be beneficial in protecting CAFO workers against chronic lung disease, who we previously showed have a higher incidence of insufficient dietary zinc intake [16]. This is highly relevant because daily dietary Zn intake is required for human health and proper immune function. Despite this, nutritional deficiency remains prevalent within vulnerable populations [28,29,30,31]. In fact, ~17% of the world’s population is at risk of inadequate zinc intake [32]. Dietary Zn deficiency increases susceptibility to pathogens [33] and is associated with a higher incidence of pneumonia [34,35], while Zn supplementation has been shown to reduce this risk [36,37,38].

In general, approximately 90% of intracellular zinc is nonlabile and considered part of the permanent matrix embedded within proteins that comprise cells. In contrast, the labile zinc pool is subject to rapid depletion in the setting of dietary deficiency. This is important because it is this pool that is responsible for cell signaling, which impacts innate and adaptive immunity. Zinc deficiency leads to an increased risk of influenza A-mediated pneumonia [39]. Whereas nasal cilia motility was shown to be stimulated in vitro through the combination of zinc and calcium [40], in our case, neither ZnCl_2_ nor ZnSO_4_ had an effect on intact tracheal cilia or isolated ciliated cells. Lung epithelial cell apoptosis and barrier leaks are also increased in the context of zinc deficiency [41]. Zinc depletion degrades junction proteins, leading to a loss of cell-to-cell contact-enhanced epithelial permeability and apoptosis, as demonstrated by Knoell et al. [42]. In addition to epithelial cells, zinc has antimicrobial effects on monocytes and macrophages, with the regulation of both monocytes and the airway epithelium by zinc being mediated through ZIP8 and NF-kB signaling [14].

Chronic alcohol intake has been shown to reduce zinc levels and the functionality of alveolar macrophages, particularly phagocytic activity [43]. Although zinc serum levels were normal in humans with alcohol use disorder (AUD), alveolar macrophage intracellular zinc levels were significantly decreased [43]. Improved phagocytic function was obtained in vitro when treating these cells with zinc acetate and glutathione [43]. Chronic alcohol intake also leads to reduced clearance of lung bacterial burden, but with the supplementation of zinc, alcohol-fed rats were able to return to a normal level of bacterial clearance in their lungs [44]. Unlike lung barrier function, we observed no effect of reversing alcohol-induced ciliary dysfunction in mice using either ZnCl_2_ or ZnSO_4_. Interestingly, ZnCl_2_ was directly stimulatory when applied to isolated ciliary axonemes whose membranes were removed by detergent extraction.

It has previously been determined that zinc transporters play a role in the clearance of lung bacteria. Zip8, a zinc transporter, is induced rapidly following a bacterial infection [45]. When Zip8 KO mice were infected with *Streptococcus pneumoniae*, they showed a higher level of inflammation, morbidity, and mortality when compared to their WT counterparts [45]. This suggests that zinc alone cannot improve lung bacterial infections and that a zinc transporter is necessary to aid in the immune response as it pertains to zinc. ZinPro is zinc conjugated to the amino acids lysine and glutamine. As such, its uptake is enhanced by effective zinc transporter action, which facilitates intracellular bioavailability for zinc target responses, such as the inhibition of PKCε, a known regulator of cilia slowing. Collectively, these findings underscore the importance of zinc transporter proteins and/or improved zinc formulations that improve bioavailability in lung epithelia.

To our knowledge, this is the first report of direct in vitro inhibition by zinc of purified PKCε enzyme catalytic activity. Previously, it was shown in a tyrosine phosphatase that zinc binding can inhibit, rather than activate, the enzyme [46,47]. This activation would then require the removal of the inhibitory zinc. Although the serine–threonine-phosphorylating PKCε, as well as many other enzymes, contain zinc-finger binding regions that are responsible for protein structural conformation [48], the mechanism of the zinc inhibition of PKCε activity is unknown at this time. With regard to in situ or in vivo inhibitory mechanisms, one possibility might be zinc-mediated alterations in lipid binding. Gomez et al. [49,50] showed that zinc changes the availability of triglyceraldehydes, although this would not explain PKCε inhibition, which binds a lipid in its diacylglycerol-binding region. Miyazaki et al. showed that zinc-deficient rats overproduce lung nitric oxide when exposed to endotoxin, a significant bioactive component of swine barn dust [51]. We have previously established that the heavy S-nitrosylation of cilia proteins can desensitize their cyclic nucleotide-mediated stimulatory function [52], but the ciliostimulatory pathway is not associated with PKC. High ZnCl_2_ concentrations (>100 µM) have been shown to produce small decreases in pH in 16HBE cells [53], but these decreases would not be sufficient to inhibit PKCε in situ. Interestingly, zinc deficiency enhances hydrogen peroxide-mediated increases in apoptosis [41], while zinc supplementation blocks this injury. Because it is well established that hydrogen peroxide can stimulate PKCε, the mechanism of action may be the inhibition of this enzyme by zinc. Future studies elucidating the role of zinc binding and active site phosphate transfer inhibition will be required to understand its mechanism of action.

Workers in CAFOs have exhibited higher rates of chronic lung disease [6,7], with approximately 25% of workers exhibiting adverse respiratory health [54]. Studies have shown that the use of PPE and respiratory masks in CAFOs is relatively low [8], and this is particularly a concern for migrant workers [16,55]. We suggest that the incorporation of a multi-factorial approach that assesses dietary- and genetically induced zinc deficiency coupled with prophylactic zinc supplementation along with other measures, including proper PPE use and wearing a respirator mask, could potentially lead to a reduced risk of chronic lung diseases in CAFO workers. In addition, community engagement with regard to nutrition among these at-risk workers may positively impact public health.

## Data Availability

The original contributions presented in the study are included in the article, and further inquiries can be directed to the corresponding author. The raw data supporting the conclusions of this article will be made available by the authors upon request.
